# Interactive effects between gaze direction and facial expression on attentional resources deployment: the task instruction and context matter

**DOI:** 10.1038/srep21706

**Published:** 2016-02-22

**Authors:** Paola Ricciardelli, Luisa Lugli, Antonello Pellicano, Cristina Iani, Roberto Nicoletti

**Affiliations:** 1 Department of Psychology, University of Milano-Bicocca, Italy; 2 Department of Philosophy and Communication, University of Bologna, Italy; 3 Division for Clinical and Cognitive Sciences, Department of Neurology Medical Faculty, RWTH Aachen University, Aachen, Germany; 4 Department of Communication and Economics, University of Modena and Reggio Emilia, Italy; 5 Milan Centre for Neuroscience, Italy

## Abstract

In three experiments, we tested whether the amount of attentional resources needed to process a face displaying neutral/angry/fearful facial expressions with direct or averted gaze depends on task instructions, and face presentation. To this end, we used a Rapid Serial Visual Presentation paradigm in which participants in Experiment 1 were first explicitly asked to discriminate whether the expression of a target face (T1) with direct or averted gaze was angry or neutral, and then to judge the orientation of a landscape (T2). Experiment 2 was identical to Experiment 1 except that participants had to discriminate the gender of the face of T1 and fearful faces were also presented randomly inter-mixed within each block of trials. Experiment 3 differed from Experiment 2 only because angry and fearful faces were never presented within the same block. The findings indicated that the presence of the attentional blink (AB) for face stimuli depends on specific combinations of gaze direction and emotional facial expressions and crucially revealed that the contextual factors (e.g., explicit instruction to process the facial expression and the presence of other emotional faces) can modify and even reverse the AB, suggesting a flexible and more contextualized deployment of attentional resources in face processing.

Facial expression and gaze direction are critical components of face processing as both communicate social and affective information, and exert a strong influence on attention^1^,^2^. Indeed, sensory processing and attention can be modulated by emotion and the affective significance of stimuli. In fact, emotionally meaningful stimuli, in particular those associated with danger or threat such as fearful and angry faces, have been shown to have priority in processing^3^ and capturing attention^4^. At the same time, direct gaze is an important cue bearing social, emotional and adaptive information since it may signal friendliness or intimacy^5^ or may also be a sign of anger or hostility^6^. Direct gaze tells us that we (the observer) are the focus of the viewer’s attention therefore its fast and accurate detection is crucial for survival as well as for social interactions.

It is now well-documented that facial expression and gaze direction interact not only to signal to the perceiver the self-relevance of the seen face^7^,^8^,^9^ but also to modulate the deployment of attentional resources^10^,^11^,^12^. As regards the face’s self-relevance, Adams and Kleck^7^ showed that direct gaze enhanced the perception of approach-oriented emotions (e.g., anger). In their study angry faces were judged faster and more accurately if they displayed direct gaze respect to averted gaze, whereas averted gaze enhanced the perception of avoidance-oriented emotions (e.g., fear) making the recognition of fearful faces with averted gaze faster than fearful faces with direct gaze^13^. Indeed, the interaction between the emotional expression on a face and the direction of gaze enhances the perception of whether the person in front of us is benevolent or hostile^14^. Therefore, detecting gaze direction in the context of facial expressions is important in order to better decode the meaning of emotional face expressions and to plan subsequent actions to avoid dangerous events or approach pleasant situations (see also^7^,^15^). Furthermore, neuroimaging studies have linked the above effects to the amygdala, a brain structure important for detecting threat and producing appropriate responses^16^,^17^. The amygdala has been shown to be involved in processing both eye gaze^18^ and emotional expression^19^, hence it seems a plausible site for the integration of these two types of facial cues. Indeed, N’Diaye and colleagues^20^ demonstrated a differential effect of gaze direction for anger and fearful expressions on amygdala responses, providing support for recent proposals that the amygdala may constitute an evolved system for appraisal of self-relevance^21^.

The prioritized processing of emotions in interaction with gaze direction can be tested under conditions where the deployment of attentional resources is limited. Recently, the availability of attentional resources and the attentional demands required in the processing of different combinations of gaze direction and facial expressions have been studied using the Rapid Serial Visual Presentation (RSVP) paradigm, which measures the deployment and temporal dynamics of attentional resources^10^,^11^,^12^,^22^. In this paradigm, two targets (T1 and T2) are embedded within a stream of successive distracters, and participants have to identify both of them. When T2 is presented in close proximity to T1 (100–500 ms) participants often fail to identify T2. This phenomenon is called attentional blink (AB) and is thought to be due to the fact that attending to T1, and consolidating it into working memory, leaves little or no attentional resources for the processing of T2 (see^23^ for a review; see also^24^ for alternative interpretations). A recent study by Ricciardelli and co-workers^12^ has shown that threatening combinations of gaze direction and facial expression (e.g., angry face staring at the observer) receive a very fast and automatic processing thus sparing attentional resources. In this study participants had to decide the gender of a neutral or an angry target face with direct or averted gaze (T1) and then to judge the orientation of a target picture of a landscape (T2), following the face at different time intervals. The results showed no attentional blink effect (i.e., no deterioration in T2 accuracy) only when T1 was an angry face with direct gaze, whereas the AB effect was present for angry faces with averted gaze, or neutral faces with either averted or direct gaze. Further evidence in this direction comes from a study by Milders and co-workers^11^. In an AB task, these authors asked participants to judge the gender of a neutral face presented in T1 and indicate whether they had seen a second face (T2) displaying an angry, fearful or happy expression with either a direct or an averted gaze. As well as finding that angry faces with direct gaze in T2 were detected more frequently than angry faces with averted gaze, it was found that fearful faces presented in T2 were detected more frequently when they displayed an averted gaze than when they displayed a direct gaze, thus complementing the results found in the study by Ricciardelli *et al.*^12^ in which only neutral and angry facial expressions were combined with direct and averted gaze.

Crucially for the present study, both the results found by Ricciardelli *et al.*^12^ and Midlers *et al.*^11^ were discussed in the framework of the appraisal theory proposed by Sander and collegues^14^. According to these authors, in fact, decoding facial expression implies a global appraisal of the face which takes into account all the observable cues in the face that convey mental states. Since gaze direction is a critical cue for inferring the focus of attention, when combined with facial expression it indicates to the perceiver the relevance of the face for his/her own needs, goals, or well-being^14^. That is, the affective meaning of a face is not fixed but is a function of the appraisal made by the observer on the basis of gaze direction and facial expression. This is why, for example, for survival an angry face looking straight at the observer signals a threat since the direct gaze indicates impending aggression towards the observer while a fearful face with averted gaze signals where in the environment a threat may come from. Thus, more generally and in line with the assumption in appraisal theories that an emotional stimulus involves “changes in a number of organismic subsystems or components”^25^, the affective meaning of the face may also be based on the evaluation of the environment and the observer-environment interaction and may depend on several contextual factors (or cues). These contextual factors may affect the allocation of attentional resources and/or change the perceived affective meaning of the seen face. More specifically, two specific contextual factors have not been taken into account yet which may change the appraisal of the face: the top-down allocation of attentional resources (i.e., the task requirement) and the specific (or contingent) situation in which the stimuli are seen.

First, the effects of task instructions on the processing of different combinations of gaze direction and facial expression have not been addressed systematically. Specifically, it is still unknown whether they can bias the allocation of attentional resources required for processing emotional faces with direct or averted gaze. Current theories of spatial and divided attention typically propose that selection can flexibly adapt to current task demands^26^, thus one can reasonably expect a degree of flexibility also in the selectivity of attention over time. Recently, the effects of task instructions have been reported in an ERP study investigating the time-course of the interaction between face gender and facial expression when this information was or was not task-relevant^27^. The results obtained showed that task instructions modulated several ERP components providing evidence that the integration and the interaction of different facial components (gender and expression) vary as a function of the task. It is worth mentioning that using explicit or implicit task instructions for processing facial expressions could lead to different results. For example, in contrast to Milders *et al.*’s^11^ and to Ricciardelli *et al.*’s^12^ study, in which the processing of the facial expression was implicit since participants were instructed to discriminate the gender of the face appearing in T1, de Jong and colleagues^10^ explicitly asked participants to identify the emotional expression of the presented face. The latter found that the attentional blink was relatively large when angry faces were presented as T1, thus suggesting that a potential social threat holds attention. It may well be the case that the task requirements biased the deployment of attentional resources per se, unbalancing the top-down allocation of attentional resources towards the emotional facial expression and resulting in an augmented allocation of attention to threatening faces. Therefore, in the light of the evidence reported above it could well be the case that also the perceived threatening value of a face stimulus, and thus the attentional demands required to process it, may vary depending on the task instructions.

Second, there is recent evidence that the ability to automatically orient our attention to socially relevant stimuli can be modulated by the specific situation in which we see them. For example, it has been shown that gaze following (i.e., the tendency to follow somebody else’s gaze direction) is not always an obligatory reflex but is subject to some sort of evaluation of the context and, if needed, it can be somehow inhibited by the observer^28^,^29^,^30^,^31^,^32^. Moreover, although there is evidence that the context in which a face appears exerts an influence on the perception and recognition of facial expressions^33^, what is unknown is whether it can also modulate how attentional resources are allocated for the processing of faces displaying different combinations of gaze direction and facial expression. Recently, it has been shown that the processing of an emotional face, and a face in general, is not an encapsulated process since it can be influenced by several situational or contextual cues^34^,^35^. In this respect, and particularly relevant to the aim of the present study, gaze direction is probably the most powerful *contextual cue*, since, as mentioned above, it best defines the threatening value of an emotional facial expression^20^,^36^. However, gaze is not the only contextual cue which contributes to the evaluation of a seen face influencing its processing. In everyday life, in fact, a frequent external contextual cue providing information for face appraisal is the presence, or appearance, of a second face. In a dynamic and changing environment like the one in which we live in, we respond to people also on the basis of the contingent situation we are experiencing. Indeed, recent evidence has shown that the presence of other emotional faces can play a role in processing the valence of a seen face^36^,^37^. This suggests that during face processing external contextual cues can be integrated with facial features (e.g., emotional expression) possibly through an automatic and mandatory process^38^, which may take place at a very early processing stage. Such integration may influence face categorization and the perceived significance and value of the seen face, ultimately influencing the allocation of attentional resources devoted to its processing. The role of external or situational context in the processing of different combinations of gaze direction and facial expression has not been tested yet but it would be informative on the way in which these two facial cues are integrated and processed.

Given the considerations reported above, the aim of the present study was twofold. First, we sought to replicate and extend the results of our previous study^12^ on the effect of gaze direction and facial expression on attention deployment by including fearful expressions. Specifically, by combining different gaze directions (direct *vs*. averted) with neutral, angry, or fearful facial expressions we aimed to investigate whether, like angry faces with direct gaze^12^, the processing of fearful faces with diverted gaze requires little or no attentional resources, or rather, since they are perceived as moderately threatening compared to angry ones^8^, they do not receive prioritized processing. Secondly, we wanted to directly test the effect of two contextual factors that do not belong to the face per se (like gaze direction) but that can affect its relevance and the deployment of attention to it. Namely, we changed task instructions by requiring either implicit or explicit processing of the facial expression, and the specific situation in which the face stimuli were presented (i.e., with or without another emotional face within the same block of trials). To this end, in three experiments we employed the Rapid Serial Visual Presentation (RSVP) paradigm to assess the degree of attentional resources required to process the stimulus presented in T1 and the amount of resources available for the processing of the stimulus presented in T2. Specifically, Experiment 1 aimed at investigating whether explicit discrimination of the emotional expression displayed by the face presented in T1 would increase the allocation of attentional resources devoted to the processing of threatening faces (i.e., angry faces with direct gaze), thus producing a significant AB effect. In Experiment 2, as in our previous study^12^, we asked participants to discriminate the gender of a face in T1, and then to judge the orientation of a landscape presented in T2. This time, however, the face in T1, in addition to the previously tested neutral and angry expressions, could also display a fearful expression. This allowed us to assess whether or not a less threatening combination of emotional facial expression and gaze direction (i.e., a fearful face with an averted gaze) produces a significant AB when presented in T1. Finally, in Experiment 3, we tested the effect of pairing neutral faces with angry or fearful faces in separate blocks of trials. In so doing, in contrast to Experiment 2, we manipulated the *contingent situation* in which the emotional faces (i.e., angry vs. fearful faces with different combinations of gaze direction) appeared. This had the scope of reducing any potential effects of presenting two emotional faces within the same block of trials which might influence the perceived significance of the face presented in T1, and bias the attentional demands needed to process it within a single trial. Given the highly context-sensitive nature of social information processing (see^34^ for a review), and the emphasis posed by appraisal theories on gaze direction as a cue to decoding the meaning of facial expressions, our expectation was to find different effects of our manipulations on the AB effect, which we considered a proxy of the attentional demand needed to process the face presented in T1.

## Experiment 1

The procedure used in Experiment 1 was identical to that used by Ricciardelli *et al.*^12^ in which participants were required to respond to each of two pre-designated targets (T1-neutral or emotional faces with direct and averted gaze; and T2-a rotated landscape), appearing in a RSVP, that were separated by different time intervals. However, differently from Ricciardelli *et al.*^12^, in the present experiment participants were required to explicitly discriminate the emotion expressed in T1 (task instruction manipulation) and then to judge the orientation of a picture of a landscape (T2). The prediction was that, in line with previous studies^12^,^22^, the AB effect, consisting in a drop in T2 accuracy, should be found also when the face in T1 was staring at the observer and displaying an angry expression. This is because the explicit instruction to discriminate the emotional expression of the face would direct and hold attention on T1-targets. Specifically, if the explicit request to discriminate the facial expression biases, via top-down mechanisms, the allocation of attention toward T1, then this should lead to a disproportional allocation of attention to T1, this resulting in an enhanced AB effect (i.e., a decrease in accuracy on T2).

### Results and discussion

**Figure 1:**
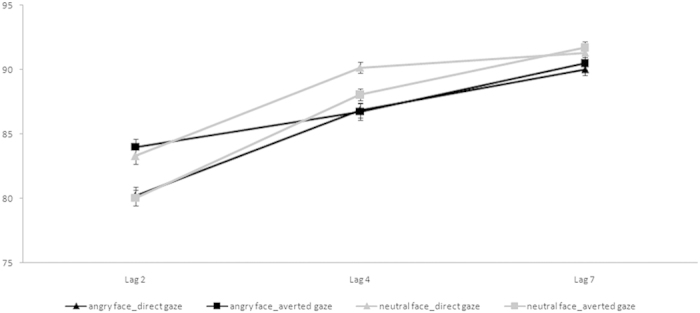
Mean accuracy (%) for angry and neutral facial expressions with averted and direct gaze directions at Lag 2, 4 and 7 in Experiment 1. Bars represent standard errors of the mean.

T2 accuracy rates were computed for T1-correct trials only (98% of total trials) and submitted to a 2 (*Facial Expression*: angry *vs*. neutral) × 2 (*Gaze Direction*: averted *vs*. direct) × 3 (*Lag*: Lag 2 *vs*. Lag 4 *vs*. Lag 7) within-subject Analysis of Variance (ANOVA). The respective data are shown in Fig. 1. Post-hoc comparisons (in this experiment and in the following ones) were performed using two-tailed t-tests with an alpha level of 0.05.

No significant main effects of *Facial Expression* [*F*(1, 45) = 3.36, *p* = 0.074, *η*_*p*_^2^ = 0.07 (i.e.,: angry = 86%, neutral = 87%], and *Gaze Direction* [*F*(1, 45) = 0.07, *p* = 0.790, *η*_*p*_^2^ = 0.002 (i.e.,: averted = 87%, direct = 87%] were found. In contrast, the main effect of *Lag* was significant [*F*(2, 90) = 60.58, *p *< 0.001, *η*_*p*_^2^ = 0.57], indicating the presence of a robust AB. Accuracy rates were 82%, 88%, and 91% for Lag 2, 4 and 7, respectively. Paired Sample T-tests showed significant differences between Lags 2 and 4 [*t*(45) = 6.75, *p *< 0.001], between Lags 2 and 7 [*t*(45)=9.31, *p *< 0.001] and between Lags 4 and 7 [*t*(45) = 4.98, *p *< 0.001].

The first-order interaction between *Facial Expression* and *Gaze Direction* was significant [*F*(1, 45) = 12.84, *p* = 0.001, *η*_*p*_^2^ = 0.22]. Paired Sample T-tests showed that for the angry facial expression accuracy was significantly lower for the direct gaze (86%) compared to the averted gaze (87%) [*t*(45) = 2.10, *p* = 0.041], whereas for the neutral facial expression accuracy was significantly lower for the averted gaze (87%) compared to the direct gaze (88%) [*t*(45) = 2.79, *p* = 0.008]. Furthermore, in the direct gaze condition accuracy was lower for the angry facial expression (86%) than for the neutral one (88%) [*t*(45) = 3.84, *p *< 0.001]. This interaction is indicative of a modulation of gaze direction on performance that varies depending on the combined facial expression.

The *Facial Expression* and *Lag* interaction was also significant [*F*(2, 90) = 3.43, *p* = 0.037, *η*_*p*_^2^ = 0.07]. Paired Sample T-tests showed that in both the angry and neutral facial expressions, accuracy increased significantly from Lag 2 to Lag 7 (i.e.: 82%, 87%, and 90%; 82%, 89%, and 92%, for the angry and neutral facial expression, respectively) [*t*_s_(45)  > 3.27, *p*_s_* *< 0.01]. Furthermore at Lag 4, T-tests showed a significant difference between angry (87%) and neutral (89%) facial expressions [*t*(45) = 2.40, *p* = 0.020], whereas at Lag 7 this difference tended to significance (90% vs. 92%) [*t*(45) = 1.88, *p* = 0.066]. This interaction revels that both neutral and angry expressions are subject to AB, therefore demand attentional resources to be processed.

Crucially, the second-order *Facial Expression* × *Gaze Direction* × *Lag* interaction was significant [*F*(2, 90) = 4.89, *p* = 0.010, *η*_*p*_^2^ = 0.10]. Paired Sample T-tests showed that in the angry expression with direct gaze, accuracy differed between all lags (80%, 87%, and 90% for Lags 2, 4 and 7, respectively) [*t*_s_(45) > 3.14, *p*_s_* *< 0.01], whereas, for the averted gaze T-tests showed significant differences between Lags 2 (84%) and 7 (90%), and between Lags 4 (87%) and 7 (90%) [*t*_s_(45)  > 3.07, *p*_s_* *< 0.05].

In the neutral expression with direct gaze, T-tests showed significant differences between Lag 2 (83%) and Lag 4 (90%), and between Lag 2 and Lag 7 (91%) [*t*_s_(45) > 5.61, *p*_s_* *< 0.001]; whereas in the neutral expression with averted gaze, accuracy differed between all lags (80%, 88%, and 92% for Lags 2, 4, and 7, respectively) [*t*_s_(45)  > 3.77, *p*_s_* *< 0.001], indicating that the processing of a neutral face with averted gaze is more demanding than that with a direct gaze.

Vertical comparisons within the lags showed that when the gaze was averted, performance was more accurate in the angry condition (84%) than in the neutral condition (80%) only at Lag 2 [*t*(45) = 3.03, *p* = 0.004]. On the other hand, when the gaze was straight performance was less accurate in the angry condition than in the neutral condition at Lag 2 (80% vs. 83%) [*t*(45) = 2.55, *p* = 0.014] and at Lag 4 (87% vs. 90%) [*t*(45) = 2.71, *p* = 0.010].

In line with previous studies^22^,^39^ this result clearly shows that, when an explicit discrimination of facial expression is required, the AB effect is present also when T1 is an angry face with direct gaze. Furthermore, the comparisons within the lags indicate that both angry direct faces and neutral averted faces require more attentional resources to be processed than angry averted and neutral direct faces since they produce a longer AB period than neutral faces with direct gaze. These findings, compared to those found in Ricciardelli *et al.*’s study^12^, speak in favor of the relevance of the task demand (i.e., implicit *vs.* explicit processing of facial expression) showing that, when observers are directly focused on the identification of specific facial expressions, the allocation of attention increases for the threatening faces.

## Experiment 2

The purpose of Experiment 2 was to extend our investigation to *fearful* facial expressions and to test whether, when combined with averted gaze, fearful faces demand little or no attention to be processed. As in our previous study^12^, in the present experiment we asked participants to discriminate the gender of the face appearing in T1 and then to judge the orientation of a landscape presented in T2, but this time the face in T1, as well as bearing a neutral or an angry expression, could also bearing a fearful facial expression. We expected that: if a fearful face with an averted gaze represents a real threat for the observer, more than a fearful face with a direct gaze^7^,^14^,^15^, then it should demand little or no attentional resources to be processed and thus, as for an angry face with direct gaze^12^, attentional resources should be available for the processing of T2, so that no decrease in accuracy on T2 should be found (i.e., reduced, or no AB in T2 task). On the contrary, if the threatening value of a fearful face with an averted gaze is much weaker than that of an angry face with direct gaze, as it does not represent a *direct* threat to the observer, then an AB effect should also emerge for targets appearing after averted gaze-fearful faces.

### Results and discussion

T2 accuracy rates were computed for T1-correct trials only (99% of total trials) and submitted to a 3 (*Facial Expression*: angry *vs*. fearful *vs*. neutral) × 2 (*Gaze Direction*: averted *vs*. direct) × 3 (*Lag*: Lag 2 *vs*. Lag 4 *vs*. Lag 7) within-subject Analysis of Variance (ANOVA).

The analysis revealed no main effect of *Facial Expression* [*F*(2, 74)* *< 1, *p* = 0.646, *η*_*p*_^2^ = 0.01] and *Gaze Direction* [*F*(1, 37)* *< 1, *p* = 0.877, *η*_*p*_^2^ = 0.001]. The main effect of *Lag* was significant [*F*(2, 74) = 20.30, *p *< 0.001, *η*_*p*_^2^ = 0.35], indicating the presence of an AB effect. Paired Sample T-tests showed more accurate performance at Lag 4 (89%) than at Lag 2 (86%) [*t*(37) = 4.99, *p *< 0.001] and no difference between Lags 4 and 7 (90%) [*t*(37) = 1.41, *p* = 0.166].

**Figure 2:**
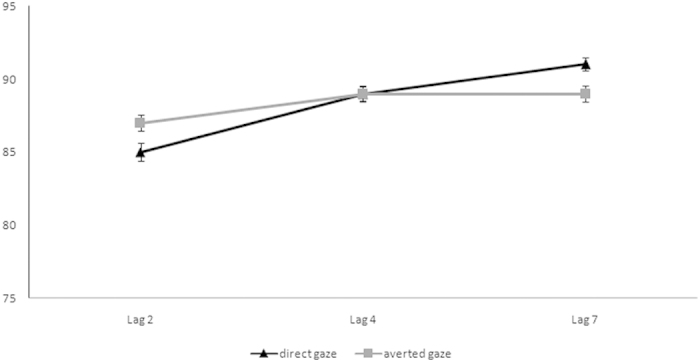
Mean accuracy (%) for direct and averted gaze directions at Lag 2, 4 and 7 in Experiment 2. Bars represent standard errors of the mean.

The first-order *Gaze Direction* × *Lag* interaction was significant [*F*(2, 74) = 4.44, *p* = 0.015, *η*_*p*_^2^ = 0.11]. Paired Sample T-tests showed that accuracy increased at Lag 7 compared to Lag 2 for both the direct gaze direction (91% vs. 85%) [*t*(37) = 6.40, *p *< 0.001], and the averted gaze direction (89% vs. 87%) [*t*(37) = 2.51, *p* = 0.017]. The difference between direct and averted gaze conditions was significant at Lag 2 [*t*(37) = 2.25, *p* = 0.031], and not significant at Lag 4 [*t*(37) = 0.32, *p* = 0.748], and Lag 7 [*t*(37) = 1.78, *p* = 0.084], indicating that the direct gaze held attention more than the averted one regardless of facial expression. No other interactions reached significance [*F*_s_* *< 1.2]. The respective data are shown in Fig. 2.

Interestingly, when a third negative facial expression, such as a fearful face, was intermixed with neutral and angry expressions, the threatening value of the angry face with direct gaze seemed to lose its prioritized processing whereas what seemed to matter was the direction of gaze, with the direct gaze calling for more attentional resources than the averted one. This suggests that the presence or absence of the AB effect elicited by emotional faces is sensitive to the context in which the face is presented. In Experiment 3 we addressed this issue more specifically.

## Experiment 3

In Experiment 3 the effect of the context was tested by blocking the presentation of the emotional faces. The experimental procedure was identical to that used in Experiment 2, with the exception that the facial emotional expressions (angry vs. fearful) were blocked within participants. Specifically, in one block we presented faces with an angry and a neutral facial expression (like in our previous study^12^), while in the other block we presented faces with a fearful and a neutral facial expression. If the context of presentation of an emotional face plays a role in the allocation of attentional resource, then a different pattern of results in terms of the AB effect should emerge between Experiments 2 and 3. Specifically, at least for angry faces, we expected to replicate the results of our previous study^12^, reporting no attentional blink effect when T1 was an angry face with direct gaze.

### Results and discussion

**Figure 3:**
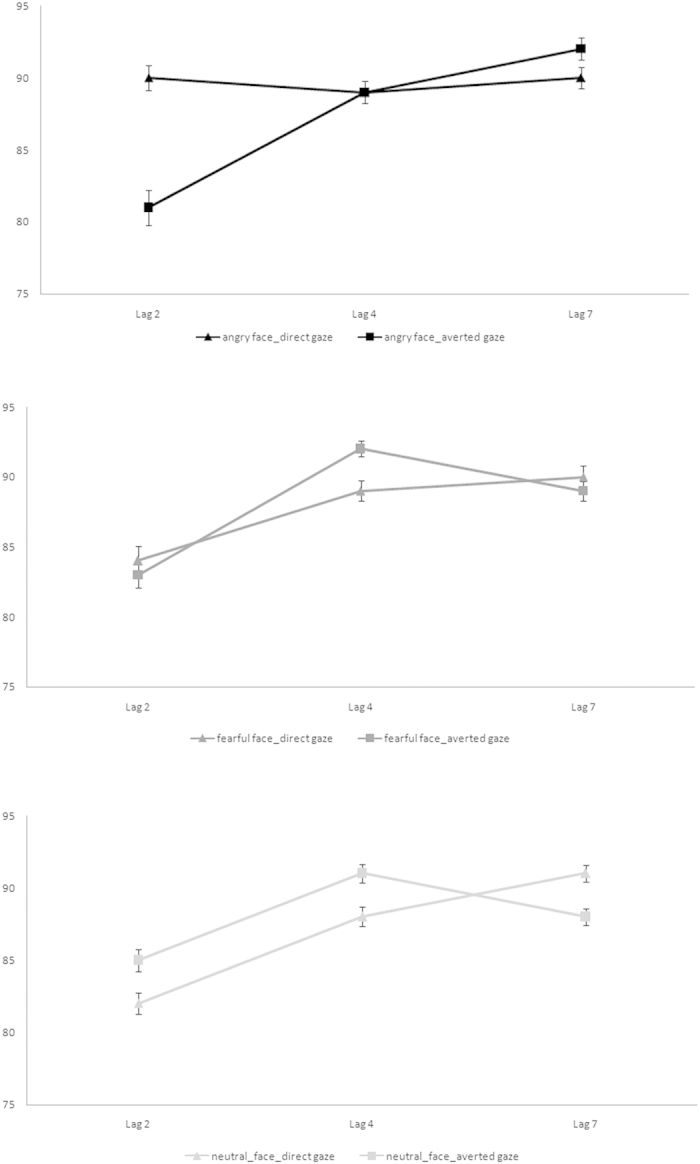
Mean accuracy (%) for angry (top panel), fearful (middle panel) and neutral (bottom panel) facial expressions with averted and direct gaze directions at Lag 2, 4 and 7 in Experiment 3. Bars represent standard errors of the mean.

T2 accuracy rates were computed for T1-correct trials only (98% of total trials) and submitted to a 3 (*Facial Expression*: angry *vs*. fearful *vs*. neutral) × 2 (*Gaze Direction*: averted *vs*. direct) × 3 (*Lag*: Lag 2 *vs*. Lag 4 *vs*. Lag 7) within-subject Analysis of Variance (ANOVA). The respective data are shown in Fig. 3.

The analysis revealed no main effect of *Facial Expression* [*F*(2, 66)* *< 1, *p* = 0.466, *η*_*p*_^2^ = 0.02] and *Gaze Direction* [*F*(1, 33)* *< 1, *p* = 0.451, *η*_*p*_^2^ = 0.02], but a significant main effect of *Lag* [*F*(2, 66) = 21.57, *p *< 0.001, *η*_*p*_^2^ = 0.40], once again indicating the presence of an AB effect. Accuracy rates were 84%, 89%, and 90% for Lag 2, 4 and 7, respectively. Paired Sample T-tests showed significant differences between Lags 2 and 4 [*t*(33) = 4.55, *p *< 0.001], and between Lags 2 and 7 [*t*(33) = 5.79, *p *< 0.001]. The difference between Lags 4 and 7 did not reach significance [*t*(33)=0.73, *p* = 0.473].

The *Gaze Direction* × *Lag* interaction was also significant [*F*(2, 66) = 5.56, *p* = 0.006, *η*_*p*_^2^ = 0.14]. Accuracy at Lags 2, 4, and 7 was 85%, 89%, and 90% for the direct gaze direction, and 83%, 90%, and 90% for the averted gaze direction, respectively. T-tests showed significant differences between Lag 2 and Lag 4 for both direct gaze [*t*(33) = 2.48, *p* = 0.018], and averted gaze condition [*t*(33) = 5.34, *p *< 0.001], whereas the difference between Lag 4 and Lag 7 was significant for the direct gaze condition [*t*(33) = 2.05, *p* = 0.048], but not for the averted one [*t*(33) = 0.84, *p* = 0.405]. Accuracy differed between Lag 2 and Lag 7 in both the direct and averted gaze conditions, [*t*_s_(33) = 4.49 and 4.96, *p*_s_* *< 0.001, respectively]. For the vertical comparisons within the lags, at Lag 2 accuracy was higher in the direct gaze (85%) than in the averted gaze condition (83%) [*t*(33) = 2.20, *p* = 0.035]. On the contrary, at Lag 4 accuracy was higher in the averted gaze (90%) than in the direct gaze condition (89%), [*t*(33) = 2.08, *p* = 0.045], indicating that the AB effect lasted longer for the direct gaze. No difference resulted at Lag 7 [*t*(33) = 0.86, *p* = 0.398]. No other first-order interaction reached significance [*F*_s_ < 2.4].

Crucially, the second-order *Facial Expression* × *Gaze Direction* × *Lag* interaction was significant [*F*(4, 132) = 5.57, *p <* 0.001, *η*_*p*_^2^ = 0.14]. For the angry facial expression - averted gaze an AB effect was found: accuracy differed between Lag 2 (81%) and Lag 4 (89%) [*t*(33) = 3.27, *p* = 0.003], and between Lag 2 and Lag 7(92%) [*t*(33) = 4.55, *p *< 0.001], whereas in line with our previous study^12^, for the direct gaze condition no significant differences (no AB effect) were found (90%, 89% and 90%, for Lag 2, 4 and 7, respectively) [*t*_s_(33) < 1, *p*_s_ > 0.401].

For the fearful facial expression - averted gaze, accuracy differed between Lag 2(83%) and Lag 4 (92%) [*t*(33) = 4.97, *p *< 0.001], and between Lag 2 and Lag 7(89%) [*t*(33) = 3.24, *p* = 0.003], indicating the presence of an AB effect for this condition. In the direct gaze fearful face condition accuracy differed between Lag 2 (84%) and Lag 4 (89%) [*t*(33) = 2.76, *p* = 0.009], and between Lag 2 and Lag 7 (90%) [*t*(33) = 2.84, *p* = 0.008], indicating also in this condition a significant AB effect (see Fig. 3).

For the neutral facial expression - averted gaze, T-tests showed significant differences between Lag 2 (85%) and Lag 4 (91%) [*t*(33) = 3.41, *p* = 0.002], between Lag 4 and Lag 7 (88%), [*t*(33) = 2.11, *p* = 0.043], but not between Lag 2 and Lag 7 [*t*(33) = 1.56, *p* = 0.129], suggesting that for neutral faces the averted gaze diverted attention somewhere else (e.g.,^40^) and away from central fixation. In the direct gaze condition, accuracy differed between Lag 2 (83%) and Lag 4 (88%) [*t*(33) = 2.30, *p* = 0.005], between Lag 4 and Lag 7 (91%) [*t*(33) = 2.07, *p* = 0.046], and between Lag 2 and Lag 7 [*t*(33) = 5.70, *p *< 0.001], indicating that direct gaze produced an AB by holding attention at fixation.

For the vertical comparisons within the lags, at Lag 2 the difference between the *angry expression*-*direct gaze* and *angry expression*-*averted gaze* was significant [*t*(33) = 5.15, *p *< 0.001], whereas it was not significant at Lags 4 and 7 [*t*_s_(33) = 0.21 and 1.07, *p*_s_ > 0.29]. The difference between the *neutral expression*-*direct gaze* and *neutral expression*-*averted gaze* was significant at Lag 7 [*t*(33) = 2.40, *p* = 0.022], and was not significant at Lags 2 and 4 [*t*_*s*_(33) = 1.48 and 1.84, *p*_s_ > 0.07].

The difference between the *fearful expression*-*direct gaze* and *fearful expression*-*averted gaze* never reached significance [*t*_s_(33) < 1.4, *p*_s_ > 0.16].

These findings are interesting for, at least, two main aspects. Firstly, using the same experimental procedure as Experiment 2, but blocking the presentation of the emotional faces, we replicated the absence of the attentional blink effect when T1 was an angry face with direct gaze^12^. This suggests that the effect of the context in which the face is presented plays a role in the allocation of attentional resources, since the threatening value of an angry face with direct gaze seemed to recover its prioritized processing when it was presented alone, compared to when it was intermixed with a fearful facial expression. Secondly, new evidence was provided as regards the fearful facial expression. More specifically, we found an AB effect for the fearful face with both direct and averted gaze direction.

## General Discussion

Our aim was to investigate whether the attentional resources needed to process the combination of direct *vs*. averted gaze with a neutral, angry, or fearful facial expression varied as a function of (1) the relevance of the face stimulus to the observer^7^,^14^,^15^, (2) the task instructions (i.e., implicit *vs.* explicit processing of facial expression) and (3) the different ways of presenting the face stimuli. This was addressed through three experiments in which a Rapid Serial Visual Presentation (RSVP) paradigm was used and the AB effect (i.e., a decrease in accuracy in the detection of T2) was taken as the indicator of the attentional demand needed to process the face presented in T1. Each experiment employed a different manipulation thought to affect the way attentional resources could be allocated. Specifically, in Experiment 1 participants were asked to explicitly discriminate the facial expression of the face presented in T1, whereas in Experiments 2 and 3 the participants were required to discriminate the gender of the facial expression presented either with or without another emotional face within the same block of trials. Our reasoning was that angry faces with direct gaze and fearful faces with averted gaze should demand less attention to be processed because, according to appraisal theories, they are evaluated by the observer as more self-relevant. In particular, angry faces with direct gaze should be perceived as more threatening, and thus as more emotional salient, and therefore modulate the deployment of attention by receiving prioritized processing. However, given the highly context-sensitive nature of social information processing, this does not imply that this appraisal effect is insensitive to other contextual factors (i.e., task instruction and the way to present the seen face). Therefore, we also expected to find a modulatory effect of these factors on the required attentional resources.

On the one hand, in line with previous studies^22^,^39^, the findings of Experiment 1 clearly showed that when the task explicitly required the discrimination of the facial expression, the AB effect was present also when an angry face with direct gaze was presented in T1. Furthermore, the comparisons within the Lags indicated that both angry faces with direct gaze and neutral faces with averted gaze require more attentional resource to be processed than angry faces with averted gaze and neutral faces with direct gaze since they produced a longer AB period than the one following neutral faces with direct gaze. These findings, taken together with those found in Ricciardelli *et al.*^12^ in which the AB effect was absent for the angry face with direct gaze when T1 task required to discriminate face gender, suggest a flexible deployment of attentional resources in face and emotion processing. In other words, the current data demonstrate that when observers are explicitly asked to focus on the identification of the specific facial expression, the allocation of attention augments for those combinations of gaze direction and facial expression which are more informative for the observer either because they represent a threat for her/him (i.e., angry faces with direct gaze) or because they signal where the other person is paying attention (i.e., neutral faces with averted gaze). This is an important result showing not only that the attentional resources can be modulated by the specific task instruction, but also that top-down and fast stimulus appraisal processes interact. In fact, it showed that when the emotional expression is task-relevant, the top-down allocation of attentional resources is more biased towards T1. This in turn holds attention on the face when it communicates important social information such as the behavioral tendency and the focus of attention of the other person as indicated by gaze direction and facial expression. This could lead to a delay in the disengagement of attention specifically from stimuli that convey threat or danger (i.e., angry face with direct gaze) or from stimuli that indicate where the person we are looking at is attending (i.e., neutral face with averted gaze). This is a new result that does not support the view that the capacity of emotional stimuli to capture (or not) attention is relatively stable or hard-wired, and consequently also relatively inflexible^41^. Recently, an alternative mechanism has been proposed: Brosch and Van Bavel^42^, through a “social identity” manipulation (i.e., in a brief learning phase participants were asked to remember the in-group or out-group affiliation of each individual face presented and then these faces were used as cues in a dot probe task) aimed at changing the attentional prioritization. They demonstrated that “emotional attention mechanisms sub-serving the selection and prioritization of relevant aspects of the environment are not static and hard-wired, but may rapidly adapt to recent changes in motivational contingencies” (*ibidem*, p.314). Our pattern of results seems to reflect this new proposal, showing that attentional deployment is flexible so as to meet the specific and contingent demands required by the specific task at hand. In fact, while in Ricciardelli *et al.*^12^ participants were required to pay attention to the face gender and no AB effect was found when angry faces with direct gaze were presented in T1, in Experiment 1 the request to focus attention on the facial expression induced an attentional prioritization change which was motivationally accommodated through a flexibly tuning/allocation of attentional resource to task relevant information.

On the other hand, the findings of Experiments 2 and 3 in which facial emotional expression was not relevant for the task suggest a substantial role of external contextual factors in attention allocation during face processing. Specifically, when fearful and angry faces with direct or averted gaze were presented randomly inter-mixed with neutral ones (Experiment 2), only an effect of gaze direction emerged, with the direct gaze inducing a larger AB effect than the averted one. However, when angry and fearful faces with direct or averted gaze were presented along with a neutral faces, but the two facial emotional expressions never co-existed within the same block (Experiment 3), the most threatening combination of facial expressions and gaze direction (angry face with direct gaze) returned to receive a prioritized processing, and once again, as in Ricciardelli *et al.*’s study^12^, no AB effect was found when it appeared in T1.

These findings are relevant for at least two reasons. First, they indicate that the processing of facial expressions is heavily influenced by contextual factors both internal and external to the face. In particular by direct gaze, a within-face feature which influences the appraisal processing of the face making it very relevant to the observer (e.g., for a review see^43^). This is particularly true in situations in which neutral facial expressions are mixed with negative valued ones (i.e., angry and fearful). In this context, angry and fearful expressions may have transferred a negative meaning to the neutral face leading to it being categorized as negative^44^,^45^ and thus giving priority of processing to all faces with direct gaze. This would explain why in Experiment 2 we found only a significant interaction between *Gaze Direction* and *Lag,* which was due to the absence of the AB effect when a face with direct gaze was presented in T1. Such a result suggests a primacy of direct gaze over facial expression when different facial expressions are presented randomly within the same blocks: the difference between direct and averted gaze conditions in the specific Lags indicated that direct gaze held attention more than averted gaze regardless of the facial expression. The salience of the eyes as perceptual features within the face has been well demonstrated behaviorally, psychophysically and neurophysiologically^46^,^47^. Eye contact and gaze behavior in general are important aspects of the human interaction and the eye region is used as an informative signal to understand mental states of other individuals^48^,^49^ in determining the social meaning of faces, including the information regarding facial expression. The present evidence suggests that the processing of facial expressions may be fundamentally different depending on whether we interpret the meaning of the expression as relevant to us or not, supporting a strict interaction between gaze direction and facial expression^7^,^8^,^9^,^14^,^50^ which flexibly affects the amount of attentional resources devoted to face processing. In Experiment 2, the fact that the different facial expressions with direct or averted gaze were presented randomly may have rendered all the ones with direct gaze alerting or threatening due to a fast and “dirty” categorization in positive (not harmful – faces with averted gaze) vs. negative (potentially threatening – faces with direct gaze). In other words, for the observer what matters in this first categorization of the face stimulus is whether or not s/he is looked at, and thus whether or not s/he is the target of a potential threatening stimulus. In fact, eye contact with the observer can be more meaningful than the specific emotion expressed, since direct gaze provides the information needed to infer if the potential environmental threat is directly oriented to him/her. The neural substrates of this categorisation process may mainly involve the amygdala that is thought to respond to crude representations of stimuli^51^ and is engaged in basic emotion recognition or arousal processing. The amygdala is an integral part of the appraisal system that takes into account both gaze direction and facial expression in prioritizing the processing of relevant stimuli^18^,^20^,^43^,^52^. Specifically, one could argue that when the observer is inserted in a context in which s/he is presented with faces that indicated a general dangerous situation (either an angry face or a fearful face) the face feature that receives priority of processing is gaze direction since it informs the observer where the attention of the other person is oriented, and thus it is processed quickly and pre-attentively sparing attentional resources for the processing of a subsequent stimulus. Specifically, because the amydgala is where the majority of subcortical and cortical inputs converge^53^ one can speculate that it is the best candidate to play a crucial role in providing both direct and indirect top-down signals on face processing, which can influence the representation of emotional faces, especially those related to threat and the observer’s self-relevance. In other words, during face processing the eye region could be used by the amydgala to prime additional neural circuits needed to decode more detailed facial information. Prioritized processing of emotional faces (especially threatening ones) might therefore results from direct “feedback” signals imposed by amydgala on cortical pathways, potentially additive or interactive with other contextual and top-down factors (e.g., task instruction) imposed by attentional systems.

Second, in line with our explanation, the results of Experiment 3, in which the threatening stimuli (angry and fearful facial expression) were processed separately from each other, suggest a different categorisation process and face appraisal (compared to Experiment 2) which again takes into account both facial expression and gaze direction. That is, the emotional information combined with the direction of the gaze return to be prioritized and angry faces with direct gaze received automatic and prioritized processing, thus replicating our previous study^12^. Interestingly, we also extended the results of our previous study because in Experiment 3 we found an AB effect for fearful faces appearing in T1, regardless of gaze direction. In particular, the AB effect found for the fearful faces with averted gaze suggested that the threatening value of this combination of facial expression and gaze direction is weaker than that of angry face with direct gaze. It is noteworthy that the present result does not contradict the fact that in Milder *et al.*^11^ fearful faces with averted gaze were detected more frequently than fearful faces with direct gaze. First, because in their study fearful faces appeared in T2 while in the present study they appeared in T1, second because the task required to be performed was different (face detection in Milder *et al.*’s study^11^ vs. a gender discrimination task in our study), and, consequently, the attentional resources needed to perform the two tasks are different, with the detection task being easier than the discrimination task. Moreover, our present result is in line with a recent study by Stein and colleagues^54^ that demonstrated that the processing of fearful facial expressions is not always completely automatic.

Interestingly then, the results of Experiment 3 compared to those found in Experiment 2 also underline the importance of external contextual factors as a strong determinant of the way in which attentional resources are allocated to process emotional stimuli^53^. In fact, the effect of the context (i.e., the trial blocks) in which the face is presented plays a role in the allocation of attentional resources, since the threatening value of angry faces with direct gaze seemed to recover its prioritized processing when they were the only emotional faces presented in the block (Experiment 3) compared to when they were mixed with fearful faces (Experiment 2). It may well be the case that when multiple emotional facial expressions had to be processed within the same block of trials, what happened was an automatic categorisation and grouping of these facial expressions along a valence dimension (e.g., positive vs. negative; self-referential vs. not self-referential) so as to prioritize the processing and to spare attentional resources. In this respect, in Experiment 2, angry and fearful expression may have been grouped together in just one alerting/negative valence chunk which represented the great majority of the stimuli. This would have then influenced the evaluation of the neutral facial expressions^27^,^44^,^55^ that were thus perceived and categorized as negative and grouped together with angry and fearful faces. Therefore, the only dimension which could distinguish the different face stimuli was the self-referential one (i.e., direct gaze vs. averted gaze). This was reflected in the effect of gaze direction, and specifically in the fact that all faces with direct gaze received prioritized processing. On the contrary in Experiment 3, when participants were presented only with one emotional expression (either the angry or fearful expression) within each block of trials, face categorisation/evaluation could be done in a more fine-grained fashion and the angry facial expression with direct gaze returned to being processed with priority. The results from both Experiments 2 and 3 could be read in line with the self-referential mode of processing model^53^ in which stimuli that are involved in a “what is happening to me” question are also the ones that are being automatically processed. Further research is needed to address this issue more systematically. Altogether, the pattern of results obtained here adds new evidence about the effect of the contextual factors (either within-face or external to it) in the allocation of attentional resources devoted to face processing. These are important findings in agreement with theoretical approaches^56^ which maintain that facial expressions do not speak for themselves^14^,^57^ and can affect cognitive processes differently. In particular, our findings indicated that the presence of AB for face stimuli depends both on the affective significance of the face stimuli and on the interaction of different facial cues (gaze direction and expression), but can also be reversed by task instructions. In other words, different combinations of gaze direction and facial expression (e.g., angry faces with direct gaze and fearful faces with averted gaze) differ in their ability to draw attention and in the demand of attentional resources for processing. The attentional demand is likely to be the result of a combination of contextual and appraisal effects that change the priority of the processing of a seen face.

In conclusion, the present study suggests that facial expressions are not always processed in the same manner, but their processing is subject to contextual modulations lying within-face, within-perceiver, or in the environment, and it speaks in favour of a flexible and more contextualized deployment of attentional resources when processing faces.

## Methods

### Experiment 1

#### Participants

Seventy-one university students (42 females, 4 left-handed, M_age_ = 23.54, SD_age_ = 3.19, age range: 19–35 years) participated. We excluded 25 participants (35% of the total) on the basis of the following exclusion criteria (identical for all the three experiments): 8 participants who showed low motivation/engagement into the task (quantified in accuracy at the T1 task lower than 95%) and 17 participants who had accuracy at the T2 task lower than 78% (i.e., the lower-bound value in^29^). Therefore, our final sample consisted of forty-six participants (25 females, 3 left-handed, M_age_ = 23.76, SD_age_ = 2.77, age range: 19–28 years). All were naïve as to the purpose of the experiment and reported having normal or corrected-to-normal vision.

#### Ethics Statement

All the experiments included in this study were performed in accordance with the ethical standards laid down in the Declaration of Helsinki and fulfilled the ethical standard procedure recommended by the Italian Association of Psychology (AIP). They were approved by the ethical committees of the University of Milano - Bicocca and of the University of Bologna; all participants provided a written informed consent.

#### Apparatus and stimuli

Participants entered a sound-attenuated, dimly lit room and were seated with their chin placed on a rest. The stimuli were presented on a 17″ cathode-ray tube screen driven by a 2.08 GHz processor computer and located at a 60-cm distance from the observer. Stimulus presentation and response registration were controlled by the E-Prime 2 software.

The stimuli were grayscale photographs of 4 different Caucasian faces and 14 landscapes. All of the stimuli were cropped to remove extraneous background and measured 4.1° × 5.9°. The landscape photographs were selected from open-access internet images. Face stimuli were selected from the Karolinska Directed Emotional Faces^58^ and depicted 2 women and 2 men (all unknown to participants) showing both a neutral and angry facial expression with direct gaze direction.

Two new versions of each photograph were created using Adobe Photoshop software: one version gazing left and the other gazing right, resulting in twenty-four pictures (4 different individuals × 2 facial expressions × 3 gaze directions). These face stimuli were selected on the basis of a rating analysis to assess whether the neutral and angry emotions were actually conveyed^12^. Landscapes were drawn from an additional pool of 50 landscape photographs that had been rotated 90° either clockwise or counterclockwise (while maintaining the same proportions as the non-rotated images), resulting in forty-two pictures (14 different landscapes × 3 orientations).

#### Procedure

**Figure 4:**
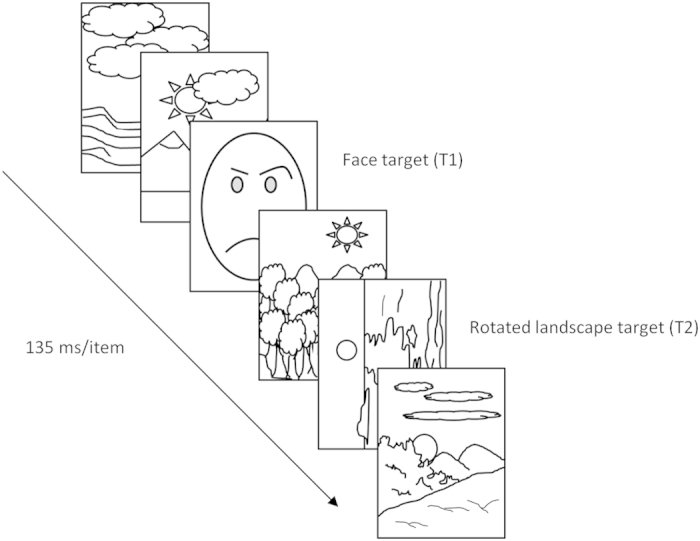
Schematic illustration of a rapid serial visual presentation (RSVP) trial. Here, the face target (T1) is an angry face with direct gaze and the rotated landscape target (T2) appears one item after it (Lag 2). In this example, due to the open access nature of the journal and copyright restrictions, the face target, the landscapes, and the rotated landscape target are represented by schematic drawings. In the actual procedure real photographs of a face and landscapes were presented instead of schematic drawings. L.L. made these drawings and prepared this figure. In Experiments 2 and 3 the face appearing in T1 could be either an angry or a fearful face with direct or averted gaze. See the text for further details.

The trials consisted of a RSVP of 16 images, each presented centrally for 135 ms. Except for two images, all were upright landscape photos. These two images consisted of angry or neutral versions of the faces, with direct or averted (half to the left and half to the right) gaze direction (T1) and the rotated (90 degrees to the right or to the left) landscape target (T2) stimulus. Depending on the trial, at the beginning of the sequence a number of blank images were presented in place of the upright landscape images, so that the number of visible items preceding T1 randomly varied from 4 to 7. T2 was presented one, three or six items after T1 (Lag 2, 4, 7, respectively) (see Fig. 4). Accordingly, the SOAs between T1 and T2 were 270 ms (Lag 2), 540 ms (Lag 4), and 945 ms (Lag 7).

The participants initiated each trial by pressing the space bar. At the end of the trial, a first question appeared on the screen prompting participants to discriminate the facial expression of T1 by pressing the F-key, labelled with a tag “N” (for the Italian word “neutro” meaning “neutral”) and the K-key, labelled with a tag “R” (for the Italian word “rabbia” meaning “anger”). A second question was then presented requiring the participants to distinguish the orientation of the T2 landscape by pressing the M-key, labelled with a tag “O” (clockwise) and the V-key, labelled with a tag “A” (anti-clockwise). Subsequently, a feedback on accuracy for both responses was given.

The instructions emphasized that the speed of responses was not recorded, and stressed response accuracy^59^. We did not record RTs because conventionally in the AB paradigm T2 performance is assessed using accuracy as the dependent measure. The experiment comprised 384 trials split into two blocks of 192 trials. Participants were allowed to take a short break between the blocks.

### Experiment 2

#### Participants

Forty-eight new students from the same pool as the previous experiment (23 female, 6 left handed, M_age_ = 21.63, SD_age_ = 6.77, age range: 18–57 years) participated. Following the same exclusion criteria, we excluded 10 participants (21% of the total): 3 participants who had accuracy at the T1 task lower than 95% and 7 participants, who had accuracy at the T2 task lower than 78% were not included in the analyses. Therefore, our final sample consisted of thirty-eight participants (19 female, 5 left handed, M_age_ = 21.21, SD_age_ = 4.77, age range: 18–45 years).

#### Apparatus, stimuli and procedure

Apparatus, stimuli and procedure were the same as that of Ricciardelli *et al.*^12^. The only modification concerned the addition of a fearful facial expression, so that face stimuli now depicted 2 women and 2 men showing *neutral*, *angry*, and *fearful* facial expressions with *direct* and *averted* gaze direction. The trials resulting from the orthogonal combination of face expression and gaze direction were randomized within a single block and across the whole experiment.

The participants were instructed to discriminate T1 face by pressing the F-key, labelled with a tag “F” (female) and the K-key, labelled with a tag “M” (male). A second question was then presented requiring the participants to distinguish the orientation of the T2 landscape by pressing the M-key, labelled with a tag “O” (clockwise) and the V-key, labelled with a tag “A” (anti-clockwise). Subsequently, a feedback on accuracy for both responses was given. The instructions emphasized that the speed of responses was not recorded, and stressed response accuracy. The experiment comprised 576 trials split into three blocks of 192 trials. Participants were allowed to take a short break between the blocks.

### Experiment 3

#### Participants

Forty-four new students from the same pool of the previous experiments (33 female, all right-handed, M_age_ = 23.95, SD_age_ = 7.35, age range: 19–52 years) participated. Following the same exclusion criteria, we excluded 10 participants (23% of the total): 1 participant who had accuracy at the T1 task lower than 95% and 9 participants, who had accuracy at the T2 task lower than 78% were not included in the analyses. Therefore, our final sample consisted of thirty-four participants (25 female, M_age_ = 24.56, SD_age_ = 8.20, age range: 19–52 years).

#### Apparatus, stimuli and procedure

Apparatus, stimuli and procedure were the same as those of Experiment 2. The only modification concerned the presentation of the stimuli which were now presented within two different blocks. We used a blocked design consisting of 384 trials split into two blocks of 192 trials each. Each block presented the pictures showing one emotional facial expression (i.e. either an angry or fearful facial expression), so that for example, in the first block the angry facial expression was compared to the neutral one and, in the second block, the fearful facial expression was compared to the neutral one. The order of the two blocks was counterbalanced between participants. Instructions, as in Experiment 2, required participants to discriminate the gender of the T1 face, and the orientation of the T2 landscape.

## Additional Information

**How to cite this article**: Ricciardelli, P. *et al.* Interactive effects between gaze direction and facial expression on attentional resources deployment: the task instruction and context matter. *Sci. Rep.* **6**, 21706; doi: 10.1038/srep21706 (2016).
